# Loss of CHOP Prevents Joint Degeneration and Pain in a Mouse Model of Pseudoachondroplasia

**DOI:** 10.3390/ijms26010016

**Published:** 2024-12-24

**Authors:** Jacqueline T. Hecht, Alka C. Veerisetty, Mohammad G. Hossain, Debabrata Patra, Michele Carrer, Frankie Chiu, Dorde Relic, Paymaan Jafar-nejad, Karen L. Posey

**Affiliations:** 1 Department of Pediatrics, McGovern Medical School UTHealth, Houston, TX 77030, USA; jacqueline.t.hecht@uth.tmc.edu (J.T.H.); alka.veerisetty@uth.tmc.edu (A.C.V.); mohammad.g.hossain@uth.tmc.edu (M.G.H.); frankie.chiu@uth.tmc.edu (F.C.); 2 School of Dentistry, The University of Texas Health Science Center at Houston (UTHealth), Houston, TX 77030, USA; 3 Department of Developmental Biology, Washington University School of Medicine, St. Louis, MO 63110, USA; debabratapatra@wustl.edu; 4 Ionis Pharmaceuticals, Inc., Carlsbad, CA 92010, USA; mcarrer@ionis.com (M.C.); drelic@ionis.com (D.R.); pjafar-nejad@ionis.com (P.J.)

**Keywords:** cartilage oligomeric matrix protein, COMP, autophagy, ASO, CHOP, chondrocyte, ER stress, joint degeneration

## Abstract

Pseudoachondroplasia (PSACH), a severe dwarfing condition characterized by impaired skeletal growth and early joint degeneration, results from mutations in cartilage oligomeric matrix protein (COMP). These mutations disrupt normal protein folding, leading to the accumulation of misfolded COMP in chondrocytes. The MT-COMP mouse is a murine model of PSACH that expresses D469del human COMP in response to doxycycline and replicates the PSACH chondrocyte and clinical pathology. The basis for the mutant-COMP pathology involves endoplasmic reticulum (ER) stress signaling through the PERK/eIF2α/CHOP pathway. C/EBP homologous protein (CHOP), in conjunction with a TNFα inflammatory process, upregulates mTORC1, hindering autophagy clearance of mutant COMP protein. Life-long joint pain/degeneration diminishes quality of life, and treatments other than joint replacements are urgently needed. To assess whether molecules that reduce CHOP activity should be considered as a potential treatment for PSACH, we evaluated MT-COMP mice with 50% CHOP (MT-COMP/CHOP^+/−^), antisense oligonucleotide (ASO)-mediated CHOP knockdown, and complete CHOP ablation (MT-COMP/CHOP^−/−^). While earlier studies demonstrated that loss of CHOP in MT-COMP mice reduced intracellular retention, inflammation, and growth plate chondrocyte death, we now show that it did not normalize limb growth. ASO treatment reduced CHOP mRNA by approximately 60%, as measured by RT-qPCR, but did not improve limb length similar to MT-COMP/CHOP^+/−^. Interestingly, both 50% genetic reduction and complete loss of CHOP alleviated pain, while total ablation of CHOP in MT-COMP mice was necessary to preserve joint health. These results indicate that (1) CHOP reduction therapy is not an effective strategy for improving limb length and (2) pain and chondrocyte pathology are more responsive to intervention than the prevention of joint damage.

## 1. Introduction

COMP, a non-collagenous extracellular matrix (ECM) pentameric glycoprotein, plays a role in collagen fibril assembly by bringing collagen monomers in close proximity to each other [1]. Additionally, it binds to multiple ECM proteins, contributing to the enhancement of cartilage integrity [2,3,4,5,6,7,8]. Significant efforts have been devoted to identifying how mutations in COMP lead to the phenotypic manifestations of pseudoachondroplasia (PSACH), a well-characterized dwarfing condition [9]. A defining characteristic of PSACH is its marked disproportionate short stature, with the adult height approximately that of an average stature 6-year-old [9,10,11,12]. Other features include a waddling gait, attractive angular face, extreme joint laxity, and shortened fingers and toes [9,13]. The most debilitating aspect of PSACH is the severe chronic joint pain and early joint degeneration, often necessitating joint replacements late in the second decade of life. Importantly, while joint replacements can alleviate pain in specific joints, not all joints are replaceable, but all PSACH joints are affected. Current treatments, primarily nonsteroidal anti-inflammatory drugs (NSAIDs) and joint replacements, offer some relief, but they are often insufficient to manage chronic pain effectively [9,11].

The mutant (MT)-COMP mouse was generated with the common D469del mutation that causes 30% of PSACH cases and recapitulated the clinical and growth plate pathologic findings [14,15,16,17,18]. Mutant COMP protein is expressed in response to doxycycline (DOX) induction; this leads to persistent ER stress, triggering inflammation and oxidative stress. This, in turn, exacerbates ER stress, creating a self-perpetuating pathological loop involving ER stress, inflammation, and oxidative stress processes [18,19,20,21,22,23]. Our studies identified cellular stresses in MT-COMP chondrocytes associated with degeneration, including the presence of ECM degradative enzymes (MMP-13 matrix metalloproteinase 13) and blockage of autophagy (pS6 phosphorylated S6; LC3 microtubule-associated protein 1A/1B-light chain 3 positive vesicles) [16,24,25]. ER stress protein CHOP and TNFα jointly increased the mammalian target of rapamycin complex 1 (mTORC1) signaling [16,24,25]. Elevated mTORC1 signaling favors protein expression over autophagy (macro-autophagy), a process needed to clear misfolded mutant-COMP protein, leading to an inability to restore ER homeostasis in chondrocytes. This build-up of ECM inside chondrocytes explains the massive retention of lamellar material in growth plate chondrocytes that led to the classification of PSACH as an ER storage disorder [2,26,27,28,29]. This accumulation forms an intracellular matrix resistant to degradation by cellular machinery, eventually overwhelming stress-coping mechanisms and compromising chondrocyte viability, thereby depleting cells needed for long bone growth and maintenance of articular cartilage health [12,24,30,31,32,33]. Our previous work demonstrated that genetic loss of CHOP (ER stress protein) largely eliminates mutant-COMP pathology in mice, suggesting that CHOP reduction could be therapeutic for PSACH.

CHOP has both a physiological role in development/growth and a pathological role associated with chronic ER stress [34]. Persistent ER stress in the MT-COMP mice suggested that CHOP plays a pathological role in the mutant-COMP pathology. Indeed, CHOP levels were elevated in MT-COMP mice in the growth plate and the articular chondrocytes at 4 weeks and 12 weeks, respectively [16,31,35]. CHOP’s role in the mutant-COMP pathology was defined using MT-COMP/CHOP^−/−^ mice generated by crossing the MT-COMP mouse onto a CHOP-null background (Ddit3 null). We showed that the loss of CHOP disrupted downstream signaling of the unfolded protein response, relieving intracellular retention of COMP, cell death, and inflammation (YM1, also known as chitinase 3-like 3 (CHI3L3)) in growth plate chondrocytes [31,32,35]. Moreover, articular chondrocytes from MT-COMP/CHOP^−/−^ mice at 20 weeks show less retention of COMP, reduced inflammation (TNFα, interleukin-1 beta (IL1β)), MMP13 (degenerative enzyme), senescence marker p16 cyclin-dependent kinase inhibitor 2A (p16 INK4a), and cell death and autophagy (pS6), while proteoglycan content is normalized [16]. These earlier results suggested that reducing or eliminating CHOP was a potential therapeutic approach for treating MT-COMP mice. In this work, we evaluated the genetic reduction, total ablation, and ASO knockdown of CHOP on the mutant-COMP pathology in proof-of-principle studies to define whether CHOP is a viable target for PSACH therapy.

## 2. Results

### 2.1. Loss of CHOP in MT-COMP Mice Does Not Improve Femur Length

**Figure 1:**
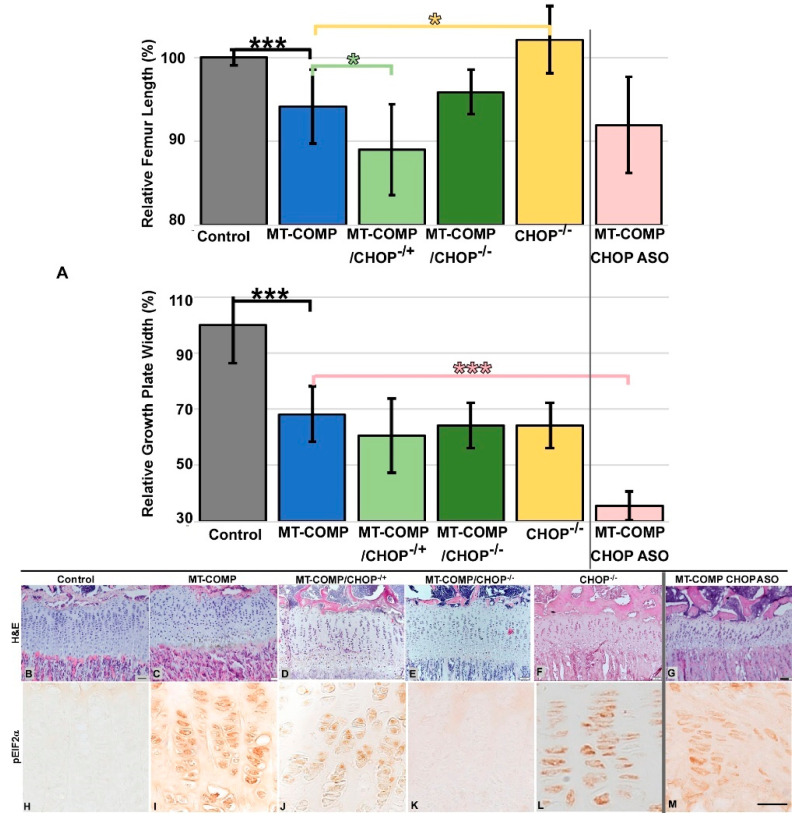
Effect of loss or reduction of CHOP on limb length and growth plate chondrocytes at 4 weeks. Femurs were collected at 4 to 5 weeks of age. (**A**) Femur lengths were measured from μCT images and growth plate widths were measured from H&E images. Each group was compared to the age-matched MT-COMP control group. Femoral length in MT-COMP was not improved in the absence of or in diminished CHOP (MT-COMP/CHOP^−/+^ and CHOP ASO-treated MT-COMP) but trended towards improvement in the absence of CHOP (MT-COMP/CHOP^−/−^). ASO-treated samples are separated by a vertical line to indicate that ASO-mediated knockdown of CHOP operates through a distinct mechanism compared to the genetic reduction of CHOP levels. Femur lengths were measured in at least 5 male mice, compared using a *t*-test. (**B**–**G**) H&E staining of control (C57BL\6), MT-COMP, MT-COMP/CHOP^−/+^ (50% CHOP), MT-COMP/CHOP^−/−^ (CHOP absent), CHOP^−/−^, and CHOP ASO-treated MT-COMP growth plates at 4 weeks is shown. (**H**–**M**) P-eIF2α immunostaining of growth plates (brown signal) is shown in the lower panel. Representative growth plates are shown from the examination of at least 8 mice of both sexes. Bar = 100 μm * = *p* < 0.05; *** = *p* < 0.0005.

To assess the effectiveness of the loss or reduction of CHOP in MT- COMP mice femoral growth, μCT imaging was used to assess femur length in MT-COMP, MT-COMP/CHOP^−/−^, MT-COMP/CHOP^−/+^, MT-COMP ASO-treated, and CHOP^−/−^ mice. In CHOP^−/−^ control mice, the absence of CHOP did not reduce femur length (Figure 1A), and consistent with previous studies, the presence of mutant-COMP significantly decreased femur length compared to C57BL\6 control mice [17] (Figure 1). The CHOP ASO was shown to be well tolerated in mice and reduces CHOP transcripts with negligible effects on other C/EBP family members [36]. ASO knockdown results in a 60% CHOP reduction in MT-COMP mice knee joints and a 99% knockdown in the liver. Moreover, growth plate width in MT-COMP/CHOP^−/−^ and MT-COMP/CHOP^−/+^ was not significantly different than MT-COMP width (Figure 1A). Neither the genetic nor ASO-mediated reduction or loss of CHOP in MT-COMP mice normalized femur length.

We next investigated the effect of reducing or eliminating CHOP in the MT-COMP growth plate. As shown in Figure 1B–G, genetic or ASO-mediated reduction or loss of CHOP appears to improve the columnar organization of growth plate chondrocytes in MT-COMP mice. The presence of p-eIF2α staining indicates that ER stress persists in CHOP ASO-treated MT-COMP (Figure 1M) and MT-COMP/CHOP^−/+^ growth plates (Figure 1J), while complete loss of CHOP (MT-COMP/CHOP^−/−^) (Figure 1K) reduces ER stress to basal control levels (Figure 1H).

### 2.2. Reduction of CHOP Partially Normalizes COMP Localization in the Growth Plate

**Figure 2:**
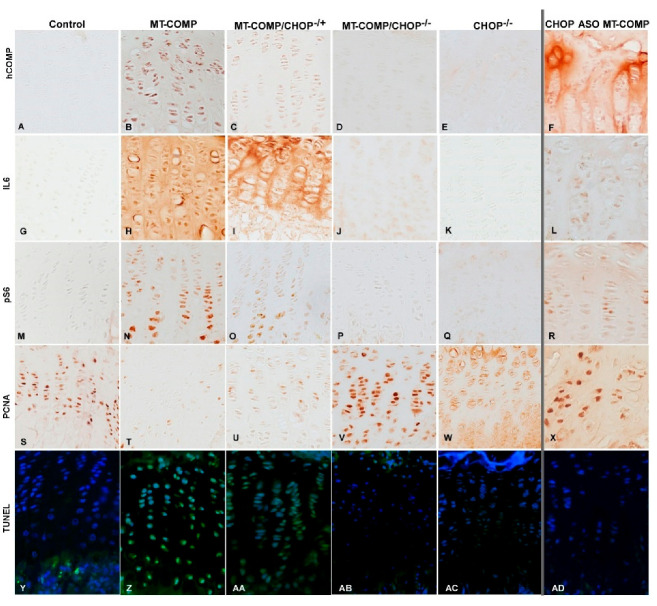
Loss or reduction of CHOP reduces ER retention of mutant COMP. Growth plates from 4-week-old control (C57BL\6), MT-COMP, MT-COMP/CHOP^−/+^ MT-COMP/CHOP^−/−^, CHOP^−/−^, and CHOP ASO-treated MT-COMP were immunostained for human-COMP (**A**–**F**), IL-6 (**G**–**L**), pS6 (**M**–**R**), PCNA (**S**–**X**) antibodies (brown signal), and apoptosis via terminal deoxynucleotidyl transferase-mediated deoxyuridine triphosphate–biotin nick-end labeling (TUNEL) (**Y**–**AD**) (TUNEL green signal; nuclei are blue). ASO-treated samples are separated by a vertical line to indicate that ASO-mediated knockdown of CHOP operates through a distinct mechanism compared to the genetic reduction of CHOP levels. The human-COMP antibody specifically recognizes human mutant-COMP expressed in MT-COMP mice in response to DOX. Controls (**A**) and CHOP^−/−^ (**E**) showed no intracellular staining for mutant-COMP compared to untreated MT-COMP growth plate chondrocytes where it was present (**B**). Representative growth plates are shown from examining at least 8 mice in both sexes. Scale bar = 50 μm.

To evaluate the mutant-COMP pathology in the growth plate of MT-COMP mice with absent or diminished CHOP, MT-COMP, MT-COMP/CHOP^−/−,^ MT-COMP/CHOP^−/+^, MT-COMP ASO-treated CHOP^−/−^, and control growth plates were assessed for intracellular retention of COMP and the presence of IL-6, pS6, PCNA, and TUNEL. Figure 2D shows that the complete elimination of CHOP in MT-COMP/CHOP^−/−^ mice effectively eliminate mutant-COMP retention comparable to the control (A) [31]. Genetic reduction of CHOP (MT-COMP/CHOP^−/+^) significantly decreases retention (Figure 2C), in contrast to the ASO treatment that moderately reduces retention while supporting some MT-COMP export to the pericellular space, the area where COMP should reside (B). Interleukin-6 (IL-6), a proinflammatory protein, was reduced with the absence or reduction of CHOP (Figure 2I,J). The absence or reduction of CHOP restored autophagy, as seen by the reduction of pS6 (Figure 2O,P). PCNA signal (indicating proliferation) is highest in controls, MT-COMP/CHOP^−/−^, and CHOP ASO-treated MT-COMP, lowest in MT-COMP, and intermediate in MT-COMP/CHOP^−/+^ groups. Only a few TUNEL-positive hypertrophic chondrocytes at the cartilage/bone junction were observed in controls, MT-COMP/CHOP^−/−^, and CHOP ASO-treated MT-COMP growth plates (Figure 2Y,AB,AD). MT-COMP growth plates exhibit numerous TUNEL-positive cells throughout all zones, whereas MT-COMP/CHOP^−/+^ growth plates have fewer overall (Figure 2Z,AA).

### 2.3. Joint Health Is Preserved with Loss of CHOP

To assess whether the absence or reduction of CHOP in MT-COMP mice mitigates early joint degeneration, histological evaluation of joint damage was performed. As described in the methods, joint degeneration was evaluated using a 0–3 scoring system to quantify degenerative alterations observed in safranin-O-stained sections of MT-COMP mice knee joints, which were not detectable with the OARSI method [37,38]. Scoring included four specific regions: (1) cartilage/bone degeneration, (2) proteoglycan loss in the tibia, (3) proteoglycan loss in the femur, and (4) the presence of synovitis. The joint degeneration score represents the sum of the scores in these four regions, with a maximum possible score of 12. Early degenerative changes were assessed in the knee joint, including synovitis, meniscus damage, and loss of proteoglycans in the articular cartilage.

**Figure 3:**
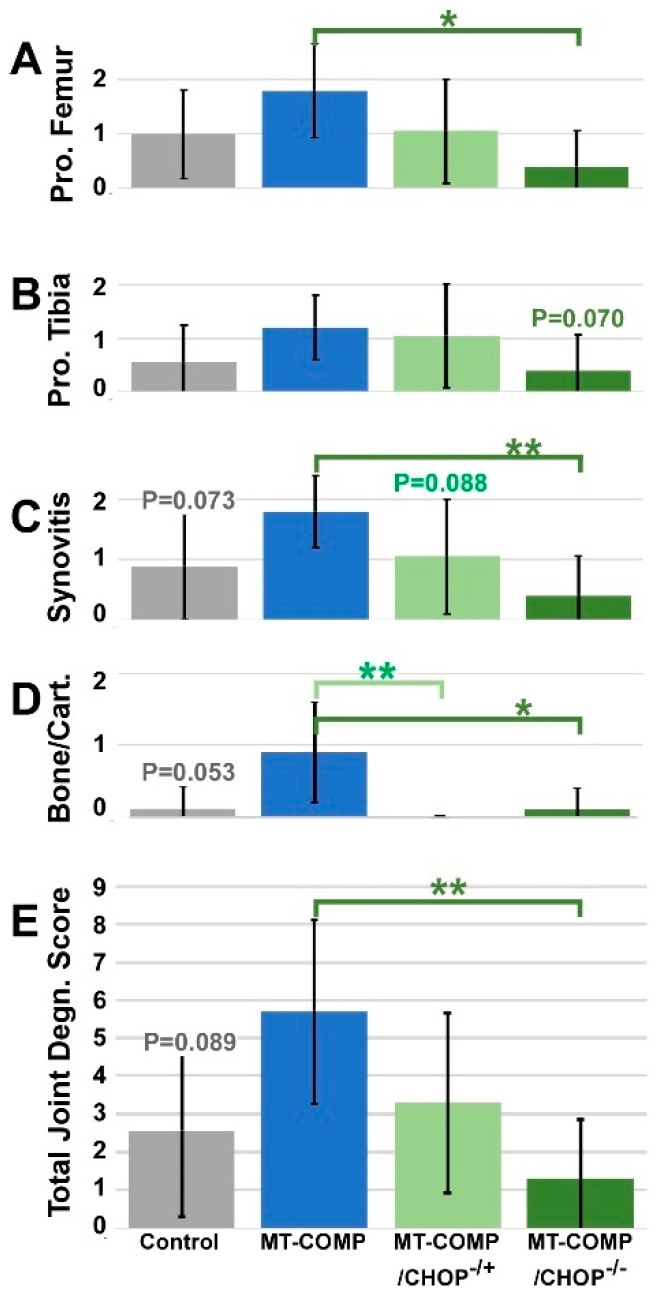
MT-COMP/CHOP^−/−^ mice do not exhibit joint degeneration at 20 weeks. Four joint health parameters were assessed in control (gray bars), MT-COMP (blue bars), MT-COMP/CHOP^−/+^ (light green bars), and MT-COMP/CHOP^−/−^ (dark green bars) joints: proteoglycan levels in femoral articular cartilage (**A**) and tibia articular cartilage (**B**), synovitis (**C**), and bone/cartilage damage (**D**). The sum of the scores for each group is shown in panel (**E**). These assessments were conducted on a minimum of 10 male mice per group. Statistical analysis was performed using the Kruskal–Wallis test with post-hoc Dunn Test with Holm-adjusted *p*-values; * indicates *p* < 0.05; ** indicates *p* < 0.005; *p*-values between 0.05–0.1 are listed. All groups were compared to MT-COMP.

Figure 3 shows the individual and total joint degeneration scores from control, MT-COMP, MT-COMP/CHOP^−/+^, and MT-COMP/CHOP^−/−^ mice. All scores were compared to MT-COMP control mice at 20 weeks. MT-COMP joints trended towards higher scores in synovitis and bone/cartilage damage, and the total score was comparable to controls, with P values approaching significance. The total joint degeneration score for MT-COMP/CHOP^−/−^ was significantly lower than that of MT-COMP mice. This reduction primarily resulted from less femoral proteoglycan, synovitis, and bone/cartilage damage (Figure 3). Although the total degeneration score for MT-COMP/CHOP^−/+^ showed a decreasing trend, only the bone/cartilage damage score was significantly different compared to the MT-COMP mice score (Figure 3).

### 2.4. Pain Mitigation Is Associated with the Absence or Reduction of CHOP in MT-COMP Mice

Changes in grooming efficiency were used here as an indicator of pain [38]. Grooming efficiency was assessed by applying a non-toxic fluorescent dye to the back of the neck between the ears, and after 4 h, the removal of the dye was evaluated as described in the methods [38].

**Figure 4:**
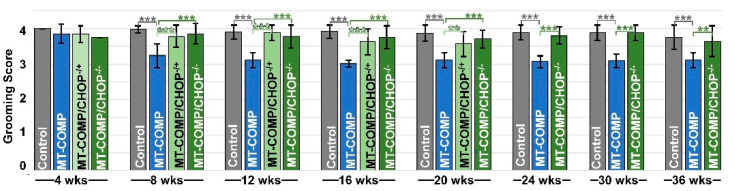
Pain is reduced with the absence or reduction of CHOP in MT-COMP mice. Grooming was used as a proxy for pain assessment by measuring the efficiency of fluorescent dye removal from the fur. The higher score indicates better dye elimination (maximum score = 5). All male mice received DOX from birth until grooming was evaluated at 4, 8, 12, 16, 20, 24, 30, and 36 weeks in control C57BL\6 (Control) and MT-COMP, MT-COMP/CHOP^−/+^, and MT-COMP/CHOP^−/−^ mice. All groups were compared to MT-COMP mice. The average grooming scores were analyzed with Kruskal–-Wallis with a post-hoc Dwass–Steel–Critchlow–Fligner (DSCF) significant pairwise test between the control and each group, and significant differences are shown by asterisks. For more information, refer to Table 1. These assessments were conducted on a minimum of 10 male mice per group. The standard deviation is shown by error bars. (Abbreviations: weeks = wks). ** *p* < 0.05; *** *p* < 0.0005.

MT-COMP mice exhibited significantly lower grooming scores than controls at 8 weeks and later ages (Figure 4). Figure 4 shows no statical difference at 4 weeks, suggesting that the onset of pain occurs between 4 to 8 weeks. Grooming scores of MT-COMP mice are lower than those of MT-COMP/CHOP^−/+^ and MT-COMP/CHOP^−/−^ mice associated with the absence or reduction of CHOP. *p*-values of comparisons are shown in Table 1.

### 2.5. Articular Chondrocyte Death and Markers of Pathologic Processes That Lead to Cell Death Are Reduced in the Absence or Reduction of CHOP

**Figure 5:**
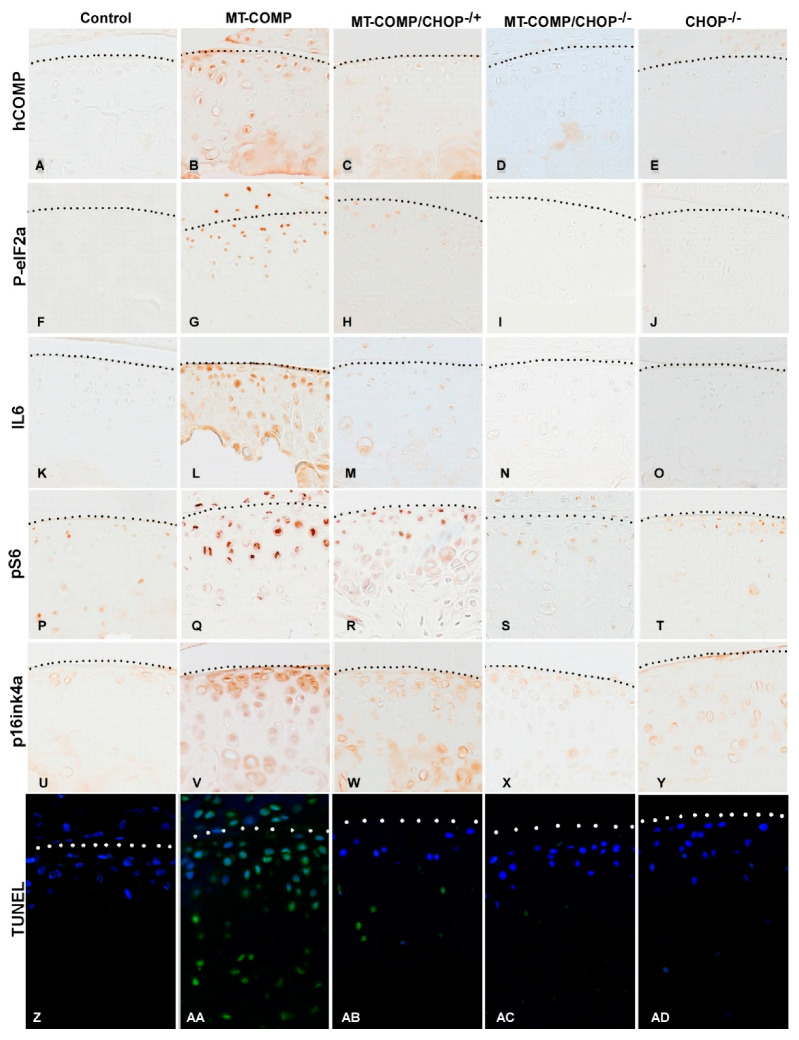
Articular cartilage chondrocyte ER stress is reduced with the absence or reduction of CHOP in MT-COMP mice. Articular cartilage from control (C57BL\6), MT-COMP, MT-COMP/CHOP^−/+^ (reduced CHOP), and MT-COMP/CHOP^−/−^ (absent CHOP) groups was immunostained for human-COMP (**A**–**E**), P-eIF2α (**F**–**J**), IL-6 (**K**–**O**), pS6 (**P**–**T**), p16INK4a (**U**–**Y**) (brown signal) and TUNEL (green signal with blue nuclei) (**Z**–**AD**) in 20-week-old mice. Representative growth plates from at least 8 mice (both sexes). Dotted line denotes the top of the articular cartilage; bar = 50 μm.

MT-COMP articular chondrocytes retained intracellular mutant-COMP and, in the absence/reduction of CHOP, had decreased retention (Figure 5). The ER stress marker, P-eIF2α (upstream of CHOP), was markedly reduced in MT-COMP/CHOP^−/−^ articular chondrocytes compared to MT-COMP controls (Figure 5F–J). Interestingly, the level of ER stress (P-eIF2α signal) appears to be much lower in the articular chondrocytes than in growth plate chondrocytes of MT-COMP mice (Figure 2 and Figure 5). As shown in Figure 5, the absence of CHOP in MT-COMP mice (MT-COMP/CHOP^−/−^) leads to the normalization of P-eIF2α, IL-6 inflammation, autophagy (pS6), and senescence (p16INK2a). The number of TUNEL-positive articular chondrocytes is lowest in MT-COMP/CHOP^−/−^, control, and CHOP^−/−^, and moderate in MT-COMP/CHOP^−/+^.

### 2.6. Loss or Reduction of CHOP in MT-COMP Lessens Inflammation and Downstream Molecules in Articular Chondrocytes

**Figure 6:**
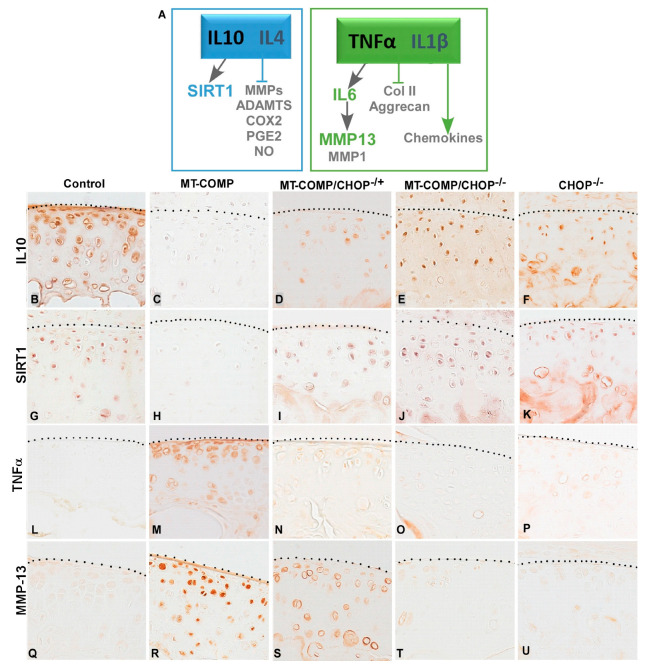
Articular chondrocytes show dampened degradation in the absence or reduction of CHOP. (**A**) Schematic showing the interaction of molecules examined in articular cartilage from control (C57BL\6), MT-COMP, MT-COMP/CHOP^−/+^ (reduced CHOP), MT-COMP/CHOP^−/−^ (absent CHOP), and CHOP^−/−^ mice were immunostained for IL-10 (**B**–**F**), SIRT1 (**G**–**K**), TNFα (**L**–**P**), and MMP13 (**Q**–**U**) antibodies at 20 weeks. Representative growth plates are shown from the examination of at least 8 mice (both sexes). Dotted line denotes the top of the articular cartilage; bar = 50 μm.

To determine whether these degenerative molecules were present in MT-COMP mice in the absence or reduction of CHOP, MT-COMP, MT-COMP/CHOP^−/−^, MT-COMP/CHOP^−/+^, CHOP^−/−^, and control articular cartilages were assessed by immunostaining. MT-COMP/CHOP^−/−^ showed an increase in both IL-10 and SIRT1 levels similar to control and CHOP^−/−^ mice and was less pronounced in the articular cartilage of MT-COMP/CHOP^−/+^ (Figure 6). Moreover, MT-COMP/CHOP^−/−^ articular cartilage had less TNFα, IL-6, and MMP-13, consistent with less cartilage/bone damage in joint degeneration scoring results (Figure 3, Figure 5 and Figure 6). The signal from MMP-13, a degenerative enzyme, was high in MT-COMP, and some was observed in MT-COMP/CHOP^−/+^, consistent with higher joint degeneration scores (Figure 3, Figure 5 and Figure 6). A 50% reduction in CHOP in MT-COMP mice improves the same components of the mutant-COMP pathology, albeit to a lesser extent than complete CHOP ablation.

## 3. Discussion

The most significant finding of this study is that genetic ablation of CHOP in MT-COMP mice effectively resolved chondrocyte stress in both the growth plate and articular cartilage, improving joint health, and was associated with eliminating pain in the PSACH mouse model. The absence of CHOP reduced ER stress and disrupted the pathological processes associated with mutant COMP, providing a potential mechanism for these therapeutic effects, consistent with other studies showing decreased osteoarthritis damage in mice lacking CHOP [39,40,41,42]. Interestingly, complete CHOP ablation was unnecessary for pain relief, as the loss of a single copy of CHOP was also associated with reduced chondrocyte pathology and pain, suggesting that therapies targeting CHOP could provide effective pain relief without full elimination, though near-complete suppression may be required to prevent joint damage. Unfortunately, despite improving most growth plate chondrocyte pathology, our antisense oligonucleotide (ASO) CHOP treatment was associated with poor health in young animals. While the ASO may still be viable in older animals, its success likely depends on achieving near-total CHOP knockdown, as a 50% reduction was insufficient to prevent joint damage.

This study took a tripartite approach to evaluate the effects of lowering CHOP levels using both genetic ablation and knockdown approaches. While reducing mutant-COMP stress with CHOP loss or reduction was expected to and did normalize chondrocyte function, it did not result in a corresponding normalization of femur length (Figure 1). Genetic approaches allowed for complete and 50% CHOP reduction, while ASO treatment achieved approximately 60% knockdown. Interestingly, despite similar reductions in CHOP levels between MT-COMP/CHOP^+/−^ and ASO-treated mice, outcomes were not identical.

CHOP ASO treatment largely had positive effects on growth plate pathology, allowing some export similar to CurQ+ (curcumin) treatment [43]. Previously, we described the complex mutant-COMP pathology [17,31,32,44,45]. Here, we evaluated MT-COMP articular cartilage for (1) intracellular retention; (2) ER stress (upstream of CHOP), inflammation, autophagy block, and senescence markers; and (3) chondrocyte death in the context of absence or reduction of CHOP in MT-COMP mice. Treatment with the CHOP ASO was superior to MT-COMP/CHOP^+/−^ in lowering IL-6 and TUNEL and increasing PCNA and equivalent autophagy blockade (pS6) and ER stress (P-eIF2α) (Figure 2). We previously demonstrated the crucial role of autophagy in clearing the ER of mutant-COMP [25], and improvements in ER stress and autophagy block are essential to efficacy [46]. In contrast, CHOP ASO knockdown therapy reduced growth plate width (Figure 1) and compromised overall fitness in mouse pups, with treated mice appearing weak, less active, and having unkempt coats compared to MT-COMP/CHOP^+/−^ mice. MT-COMP/CHOP^+/−^ and CHOP ASO treatments are inherently different with regard to mechanism and timing of CHOP reduction. Endogenous CHOP reduction in MT-COMP/CHOP^+/−^ mice starts at conception, whereas ASO treatment begins at 1 week of age and relies on endocytic uptake and RNase H-mediated mRNA degradation [47,48,49]. Before ASO administration, CHOP levels were equivalent to those in untreated MT-COMP mice. These differences may account for the ASO’s negative impact on pup health that led to the discontinuation of CHOP ASO testing. Importantly, neither CHOP reduction nor ASO treatment restored limb growth. The “gold standard” for the treatment of a dwarfing condition is an improvement in linear growth. Given that proliferation is integral to growth plate function and perhaps insufficient improvements, proliferation accounts for the thinning of growth plates with ASO treatment or CHOP reduction/elimination [50]. 

In adult mice, the CHOP ASO was well-tolerated and has been successfully used to prevent ER stress-induced liver damage in a type II diabetes mouse model [36]. While this suggests potential use in adult MT-COMP mice, it is unlikely to improve joint health, as the joint score of MT-COMP/CHOP^+/−^ mice was not significantly better than that of untreated MT-COMP mice. Taken together, these outcomes indicate that reduction of CHOP must be greater than 50% and closer to 100% to preserve joint health. The ideal treatment outcome for joint degeneration, found in PSACH and osteoarthritis, is to prevent or slow degradation and alleviate associated pain. 

Pain in PSACH, particularly in the knees and hips, significantly interferes with daily life [51]. Previously, we established that pain in MT-COMP mice is measurable using two behavioral assessments: voluntary running and grooming [38]. These indirect proxy pain assays assess changes in instinctive behaviors [38], specifically alterations in physical activity (voluntary running) and grooming. Grooming, a natural behavior, is suppressed in the presence of pain [52], which better correlates to PSACH pain rather than traditional assays like Von Frey or hot plate, which assess referred pain or allodynia [53,54]. While grooming assays are not widely used for pain evaluation in mice, they offer a rapid and consistent measure that correlates with other traditional indirect indicators of pain, such as voluntary running activity and gait analysis [16,38]. Notably, the absence of mutant COMP expression normalized grooming behavior in MT-COMP mice, suggesting this assay accurately reflects pain/stress associated with mutant-COMP expression (no DOX) [38]. To validate the association between grooming and pain in MT-COMP mice, an analgesic (ibuprofen) was administered for a 7-day period, after which, grooming was assessed. The administration of ibuprofen (0.2 mg/mL in drinking water) 7 days prior to grooming resulted in MT-COMP grooming scores comparable to those of control mice at 16 weeks of age [38]. The reduced pain in these mice correlated with lower levels of pro-inflammatory cytokines IL-6 and TNFα, and higher levels of the anti-inflammatory cytokine IL-10, suggesting that inflammation plays a key role in PSACH-associated pain. The normalization of grooming behavior with ibuprofen, a nonsteroidal anti-inflammatory drug (NSAID), further supports the conclusion that CHOP reduction decreases pain in MT-COMP mice by mitigating inflammation. Both MT-COMP/CHOP^−/−^ and MT-COMP/CHOP^+/−^ mice exhibited no pain, as measured by grooming assays suggesting that pain may be easier to manage than growth deficits.

Previous studies have shown that anti-inflammatory IL-10 blocks degenerative arthritic processes and supports cell survival through SIRT1 [55], whereas TNFα, a pro-inflammatory molecule, depresses ECM synthesis and stimulates chemokines, IL-6, and downstream MMP-13, thereby supporting cartilage break down [56]. Articular chondrocytes from MT-COMP/CHOP^−/−^ mice displayed higher IL-10 and lower TNFα levels, which influenced key molecules such as SIRT1 and MMP-13, both critical to joint health and osteoarthritis progression [55,57,58]. In MT-COMP/CHOP^−/+^ mice, the reduction of TNF and increase in IL-10 were more modest, leading to elevated MMP-13 levels, cartilage damage, and no improvement in joint health. This suggests that the same molecular pathways involved in OA-driven joint degeneration are also involved in PSACH, highlighting the potential relevance of our findings to idiopathic OA.

Overall, genetic CHOP ablation alleviates mutant COMP chondrocyte and joint pathology but does not normalize limb growth. Others have shown that CHOP is expressed in both proliferative and hypertrophic growth plate chondrocytes in mice [59], and CHOP enables chondrocyte survival cells [60]. Consistent with this, the loss of one or both copies of CHOP in MT-COMP mice did not increase growth plate width (Figure 1). We hypothesize that CHOP’s role in chondrocyte survival coupled with ER stress associated with MT-COMP explains the lack of growth recovery in the MT-COMP without one or both copies of CHOP. The MT-COMP/CHOP-null mice appear to have thin growth plates, but femur length is not statistically different from MT-COMP, likely due to the lack of ER stress driven by MT-COMP retention. Consistent with our findings that intracellular retention of misfolded mutant-COMP in the ER, in both growth plates and articular chondrocytes, is pathognomonic for PSACH and MT-COMP mice [16,18,29]. Genetic ablation is not feasible in humans with current technologies, especially since PSACH diagnosis typically occurs between 18 and 24 months. Partial CHOP knockdown reduced chondrocyte stress and provided pain relief but was not superior to NSAID therapy. Moreover, CHOP ASO treatment outcomes were mixed, similar to previous efforts with partial COMP knockdown [45], which did not rescue limb length. Our previous studies indicate that interventions in MT-COMP mice must begin by 4 weeks of age to prevent joint degeneration, suggesting that early treatment, including future drug/ASO therapies that dramatically reduce CHOP expression, would be necessary to preserve joint health. These findings do not rule out the development of more effective ASO therapies targeting articular cartilage or the use of drugs to reduce CHOP expression to preserve joint health significantly; however, they suggest that other strategies may be more effective for restoring long bone growth.

## 4. Materials and Methods

### 4.1. Bigenic Mice

MT-COMP mice were generated using two DNA-containing expression cassettes derived from plasmids, pTRE-MT-COMP (D469del-COMP mutation) and pTET-On-Col II, as previously described [18]. Mice expressed D469del-COMP protein in all tissues expressing type II collagen in the presence of doxycycline (DOX 500 ng/mL) administered through drinking water (with 5% *w*/*v* sucrose) pre- and postnatally. All mutant mice were healthy and reproduced. C57BL/6 mice were used as controls since the wild-type (WT)-COMP mice showed no phenotypic differences [18]. MT-COMP mice were generated in C57BL/6 ES cells. Moreover, C57BL/6 mice are ordered once per year from Jackson Laboratories, and mice are bred in-house and administered DOX to generate controls. Mice were housed in single-sex groups after weaning (P21) and fed standard chow (PicoLab rodent diet 20 #5053, LabDiet, St. Louis, MO, USA). The Animal Welfare Committee at the University of Texas Medical School at Houston approved these studies, and all experiments complied with the Guide for the Care and Use of Laboratory Animals: Eighth Edition, ISBN-10: 0-309-15396-4 and NIH guidelines. CHOP-null mice obtained from Jackson labs were backcrossed with C57BL/6 and then mated with MT-COMP mice to produce MT-COMP/CHOP^−/−^ and MT-COMP/CHOP^−/+^ in a C57BL/6 background [31]. MT-COMP/CHOP^−/−^ mice were mated by MT-COMP to generate MT-COMP/CHOP^−/+^ for experiments. Mice were selected from at least 5 litters for each experiment to control for biological variability.

### 4.2. Mutant COMP Induction

Doxycycline (DOX 500 ng/mL) was administered through drinking water or mother’s milk prior to weaning at 3 weeks of age with 5% *w*/*v* sucrose to increase palatability.

### 4.3. CHOP ASO Treatment

CHOP ASO from Ionis Pharmaceuticals, Carlsbad, CA, USA [36] was previously used in adult mice to prevent ER stress-induced hepatic damage resulting from type II diabetes [36]. This same ASO was administered to MT-COMP mice via intramuscular injection at 30 mg/kg three days a week from the age of 1 to 4 weeks.

### 4.4. Immunohistochemistry

Hind limbs of MT-COMP and C57BL/6 control mice (both sexes) were collected at P28, and tibial growth plates were analyzed as previously described [18]. Mouse limbs were fixed in 95% *v*/*v* ethanol for immunostaining for human COMP (Abcam, Cambridge, MA, USA, ab11056-rat 1:100), tumor necrosis factor α (TNFα) (Abcam, ab6671, 1:200), MMP13 (Abcam ab39012, 1:50), p16 INK4a (Abcam ab189034, 1:200), P-eIF2α (Cell Signaling, Danvers, MA, USA, 3597S 1:50), IL-6 (Bioss Woburn, MA, USA, bs-0781R, 1:200), SIRT1 (Abcam 32441, 1:100), pS6 (1:200 2215S rabbit polyclonal Cell Signaling Technology), PCNA staining kit (93-1143, Invitrogen, Frederick, MD, USA), IL-10 (1:200 bs-0698R Bioss Antibodies Woburn, MA, USA), or in 10% *w*/*v* formalin for terminal deoxynucleotidyl transferase-mediated deoxyuridine triphosphate–biotin nick-end labeling (TUNEL) staining. The COMP rat antibody does not cross-react with endogenous mouse COMP and only recognizes human COMP. Species-specific biotinylated secondary antibodies were incubated with each section for 1 h following streptavidin/HRP incubation and chromogen detection for visualization. At least 8 mice in each group were examined.

### 4.5. Limb Length Measurements

Hind limbs were obtained from 8 or more male mice for each group, and the soft tissue was carefully removed. The hind limb was stored in PBS and then subjected to μCT analysis (Sky Scan 1276 from Bruker, International, Billerica, MA, USA). Measurements were made from end to end on femurs using MicroView software 2.5.0 (GE Healthcare, Chicago, IL, USA), as previously described [61]. ANOVA statistics were used to compare the femoral measurements from mice limbs. Standard deviation is shown as error bars in Figure 2.

### 4.6. Grooming Assessments

Fluorescent dye (50 μL) was applied on the back of the neck equidistant between ears at time 0, and grooming efficiency was assessed 4 h later in male mice. Animals were imaged and compared to the scoring rubric previously published, with maximal grooming scoring of 5 [38,52]. The average grooming scores were analyzed with Kruskall–Wallis with the post-hoc Dwass–Steel—Critchlow–Fligner (DSCF) pairwise test. The standard deviation is represented by error bars in Figure 4; *p* value information is provided in the Table 1.

### 4.7. Joint Degeneration Scoring

The joint scoring system used in this study quantifies early degenerative changes in the proteoglycan content of articular cartilage of the femur and tibia, and the degree of synovitis and bone/cartilage damage was described previously [38,62]. This method is sensitive to early joint changes, whereas the OARSI scoring system [49] best describes end-stage joint damage. Joint degeneration scoring was performed on 5 μm sagittal sections from 10 different mice. Only sections containing both menisci were scored to ensure sections were obtained from the same area of the joint. Four areas: synovium, bone/cartilage, tibial, and femoral articular cartilage, were scored from 0 to 3 on each Safranin-O-stained section [38]. Only sections that contained both menisci were scored to ensure sections were obtained from the same area of the joint. Control and MT-COMP mice were scored using OARSI scoring, OARSI scoring + meniscus/synovium component, and the early joint pathology score previously described [38]. All scoring systems showed a trend towards a higher level of damage for the MT-COMP joints compared to controls, but only the early scoring system showed a significant difference. A score of 0 indicated normal or no damage, 1 = mild damage, 2 = moderate damage, and 3 = severe damage. Synovitis was defined as mild—increase in thickness of synovial lining and increase in stromal area, moderate—increase in stromal density, or severe—thickening of synovial lining with a further increase of stromal cellular density. A score of normal—0 indicates no synovitis detected. Bone/cartilage damage was defined as normal—the surface was smooth; mild—minor erosion of the surface; moderate—the presence of remodeling with minor erosion; or severe—major erosion. Proteoglycans of the articular cartilage of the tibia and femur were classified as normal—if staining was even through to the subchondral bone; mild—when staining was thinned; moderate—thinning of the proteoglycan-stained layer and absence of staining in some areas; or severe—the widespread loss of proteoglycan staining. Ten or more male mice per experimental group were used for each time point, providing 80–90% power to detect a minimal difference of 2 or 3 units. All scoring was performed blindly. Limb section depth, thickness, fixation, and decalcification conditions were identical. The standard deviation is shown as error bars in Figure 3. The Kruskal–Wallis test was used to evaluate the distribution of individual scored features across 4 experiment groups, and the post-hoc Dunn’s test with Holm-adjusted *p*-values for comparing MT-COMP to controls.

Ten mice per experimental group were used for each time point, providing 80–90% power to detect a minimal difference of 2 or 3 units. All scoring was performed blindly. Limb section depth, thickness, fixation, and decalcification conditions were identical. A *t*-test was used to evaluate the total joint degeneration score across six experimental groups, comparing MT-COMP to all other groups. The Kruskal–Wallis test was used to evaluate the distribution of individual scored features across six experiment groups, and the post-hoc Dunn’s test compared MT-COMP to controls.

## Figures and Tables

**Table 1:** Grooming scores were analyzed first by Kruskal–Wallis followed by post-hoc DSCF pairwise comparisons. *p* values from comparisons are shown in the table. Significant *p* values are shown in bold text.

Comparison	*p* Value at Time Points in Weeks (wks)						
	8 wk	12 wk	16 wk	20 wk	24 wk	30 wk	36 wk
Control vs. MT-COMP	**<0.001**	**<0.001**	**<0.001**	**<0.001**	**<0.001**	**<0.001**	**<0.001**
Control vs. MT-COMP/CHOP^−/−^	0.292	0.716	0.408	0.056	0.598	1.000	0.814
Control vs. MT-COMP/CHOP^−/+^	0.079	0.999	0.111	0.294	-	-	-
							
MT-COMP vs. Control	**<0.001**	**<0.001**	**<0.001**	**<0.001**	**<0.001**	**<0.001**	**<0.001**
MT-COMP vs. MT-COMP/CHOP^−/−^	**<0.001**	**<0.001**	**<0.001**	**<0.001**	**<0.001**	**<0.001**	**0.003**
MT-COMP vs. MT-COMP/CHOP^−/+^	**<0.001**	**<0.001**	**<0.001**	**0.007**	**-**	**-**	**-**

## Data Availability

Data is available upon request from the corresponding author, but data cannot be used in publications/presentations by those other than the original authors unless all original authors give express written consent.
